# The Use of Medications Approved for Alzheimer’s Disease in Autism Spectrum Disorder: A Systematic Review

**DOI:** 10.3389/fped.2014.00087

**Published:** 2014-08-22

**Authors:** Daniel A. Rossignol, Richard E. Frye

**Affiliations:** ^1^Rossignol Medical Center, Irvine, CA, USA; ^2^Arkansas Children’s Hospital Research Institute, University of Arkansas for Medical Sciences, Little Rock, AR, USA

**Keywords:** autism, Alzheimer’s disease, acetylcholinesterase inhibitors, NMDA antagonist, medications

## Abstract

Autism spectrum disorder (ASD) is a neurodevelopmental disorder that affects 1 in 68 children in the United States. Even though it is a common disorder, only two medications (risperidone and aripiprazole) are approved by the U.S. Food and Drug Administration (FDA) to treat symptoms associated with ASD. However, these medications are approved to treat irritability, which is not a core symptom of ASD. A number of novel medications, which have not been approved by the FDA to treat ASD have been used off-label in some studies to treat ASD symptoms, including medications approved for Alzheimer’s disease. Interestingly, some of these studies are high-quality, double-blind, placebo-controlled (DBPC) studies. This article systematically reviews studies published through April, 2014, which examined the use of Alzheimer’s medications in ASD, including donepezil (seven studies, two were DBPC, five out of seven reported improvements), galantamine (four studies, two were DBPC, all reported improvements), rivastigmine (one study reporting improvements), tacrine (one study reporting improvements), and memantine (nine studies, one was DBPC, eight reported improvements). An evidence-based scale was used to rank each medication. Collectively, these studies reported improvements in expressive language and communication, receptive language, social interaction, irritability, hyperactivity, attention, eye contact, emotional lability, repetitive or self-stimulatory behaviors, motor planning, disruptive behaviors, obsessive–compulsive symptoms, lethargy, overall ASD behaviors, and increased REM sleep. Reported side effects are reviewed and include irritability, gastrointestinal problems, verbal or behavioral regression, headaches, irritability, rash, tremor, sedation, vomiting, and speech problems. Both galantamine and memantine had sufficient evidence ranking for improving both core and associated symptoms of ASD. Given the lack of medications approved to treat ASD, further studies on novel medications, including Alzheimer’s disease medications, are needed.

## Background

Autism spectrum disorder (ASD) is a heterogenous neurodevelopmental disorder that is defined by behavioral observations and characterized by developmental delays in communication and social interaction and by repetitive behaviors and/or restricted interests. The most recent prevalence of ASD in the United States (U.S.) is now 1 in 68 children, including 1 in 42 boys (1). Only two medications have been approved by the U.S. Food and Drug Administration (FDA) for ASD and these medications, risperidone and aripiprazole, are indicated to treat irritability, an associated but not core symptom of ASD (2, 3). Since irritability is not a core feature of ASD, there are currently no U.S. FDA approved medications for treating the core symptoms of ASD.

A number of novel medications have been used to treat the symptoms of ASD (4, 5). Some of these medications are approved for the treatment of Alzheimer’s disease. A connection between Alzheimer’s disease and autism has been proposed by some investigators (6) and will be reviewed below. Because of the evidence of this connection, several medications approved for Alzheimer’s disease have been investigated for use in ASD. To date, five medications have been approved by the U.S. FDA for the treatment of Alzheimer’s disease: Tacrine (Cognex^®^, 1993), Donepezil (Aricept^®^, 1996), Rivastigmine (Exelon^®^, 2000), Galantamine (Razadyne^®^, 2001), and Memantine (Namenda^®^, 2003). Donepezil, galantamine, rivastigmine, and tacrine are cholinesterase inhibitors, and work by preventing the breakdown of acetylcholine. Galantamine also stimulates nicotinic cholinergic receptors and therefore can increase the release of acetylcholine (7). Memantine is distinct from these other medications and modulates glutamate neurotransmission.

Several lines of evidence have implicated abnormalities in the cholinergic system in ASD (8). First, studies examining post-mortem brain samples from individuals with ASD have reported abnormalities in the cholinergic system (9–11). Early studies compared the cholinergic system in frontal, parietal, hippocampus, and cerebellar tissue from typically developing adults to similarly aged autistic adults with intellectual disability. One of these studies found decreases in muscarinic M_1_ receptors in the parietal cortex and nicotinic receptors in the frontal and parietal cortexes, with a decrease in α_4_ and β_2_ nicotinic subtypes confirmed by immunochemistry in the parietal cortex (9). Another study found significant changes in nicotinic receptors but no significant changes in muscarinic receptors or in the presynaptic cholinergic enzyme choline acetyltransferase in the cerebellum. Consistent nicotinic receptor changes included decreases in the α_4_ receptor subtype in several types of cells including granule and Purkinje cells, as well as increases in the α_7_ receptor subtype in the granule cell layer (11). In contrast, using quantitative receptor autoradiographic studies, no changes in cholinergic receptor binding were found in the hippocampus (12). Later, a study comparing typically developing and autistic adults showed a decrease in α_7_ and β_2_ but not α_4_ nicotinic acetylcholine receptor subunits in the thalamus (10). Secondly, a positron emission tomography study reported a decrease in acetylcholinesterase activity in the bilateral fusiform gyri in ASD adults, as compared to typically developing adults, with this decrease correlated with objective scales of individual participant social disability (13). Thirdly, functional analysis of gene networks altered in individuals with ASD implicate synaptic cholinergic receptor families of genes (14) and epigenetic changes in both ASD and Rett syndrome have been linked to decreased expression of the CHRNA7 gene encoding the nicotinic receptor subtype α_7_ (15). Lastly, some studies have implicated cholinergic abnormalities in an animal model of ASD. The BTBR ASD mouse model demonstrates lower basal levels of extracellular acetylcholine in the prefrontal cortex (16) and injection of the acetylcholinesterase inhibitor donepezil into the BTBR mouse (systemically or directly) in the dorsomedial striatum, the rodent homolog of the caudate nucleus, ameliorated many core ASD behaviors (17). Thus, there is substantial support for the idea that treatments that modulate the cholinergic system might be helpful in ASD (18).

Several lines of scientific evidence have also pointed to abnormalities in glutamate metabolism in individuals with ASD as identified by imaging, genetic, and post-mortem studies. Proton magnetic resonance spectroscopy has demonstrated abnormalities in glutamate metabolism in individuals with ASD. For example, the glutamate + glutamine peak has been found to be increased in individuals with ASD as compared to controls in the auditory cortex (19), anterior cingulate cortex (20, 21), and basal ganglia (22). Another study found that the glutamate/glutamine ratio in the amygdala–hippocampal region was increased in ASD individuals as compared to controls (23), while one study found an increase in glutamate/creatine in the putamen in individuals with ASD (24). Interestingly, the magnetic resonance spectroscopy glutamate + glutamine peak in the basal ganglia of ASD individuals was correlated with a measure of impairment in social communication (22) and the glutamate/creatine ratio in the putamen was correlated with ASD symptoms (social interaction) (24). However, studies examining glutamate in frontal brain regions have demonstrated inconsistent results. For example, as compared to controls, individuals with ASD demonstrated a decrease in the GABA/glutamate ratio in the frontal cortex in one study (25) while the glutamate + glutamine peak was not different between ASD and control individuals in the prefrontal cortex in another study (22). The glutamate/creatine ratio was decreased in ASD individuals in the frontal lobes, as compared to controls, in another study (26).

Genetic studies have implicated abnormalities in ionotropic glutamate receptors in ASD. Genetic studies have associated ASD with abnormalities in subunits of *N*-methyl-d-aspartate (NMDA) (27–30), α-amino-3-hydroxy-5-methyl-4-isoxazolepropionic acid (AMPA) (31), and kainate (32, 33) receptors and the mitochondrial aspartate/glutamate carrier (34–36). Genetic syndromes that have a high prevalence of ASD features demonstrate abnormalities in glutamate neurotransmission. For example, haploinsufficiency of SHANK3, a gene that encodes postsynaptic scaffolding proteins for glutamate receptors, is characteristic of many patients with Phelan–McDermid syndrome, a genetic disorder with a high prevalence of ASD (37). Interestingly, genetic animal models of ASD have also demonstrated involvement of glutamate receptors. For example, one of the main areas of research is the Fragile X mouse model where abnormalities in metabotropic glutamate receptor subtype 5 (mGluR5) have been linked to behavioral and cognitive abnormalities that overlap symptoms associated with ASD (38).

Lastly, post-mortem studies have also implicated glutamate abnormalities. Cerebellum samples from individuals with ASD have demonstrated an increase in mRNA for AMPA 1, 2, and 3 receptors and glutamate/aspartate transporter 1 and 2, an increase in protein levels of glutamate/aspartate transporter 1 and 2 and AMPA 1 and NMDA 1 receptors, but a decrease in AMPA receptor density (39). In addition, other studies have demonstrated an increase in mGluR5 protein in the vermis (40) and superior frontal cortex (41) of children with ASD. One study reported decreased levels of kidney-type glutaminase in the anterior cingulate cortex of individuals with ASD (42), while another study demonstrated an increase in the mitochondrial aspartate/glutamate carrier in the prefrontal cortex but not in the cerebellum of individuals with ASD (43). Thus, there is substantial evidence for glutamate abnormalities in individuals with ASD, suggesting the treatments that modulate glutamate may be helpful in ASD.

Memantine is an NMDA receptor antagonist and regulates the activity of glutamate, a neurotransmitter involved in memory and learning. Normally glutamate attaches to NMDA receptors allowing calcium to then enter freely into cells. Memantine prevents this by partially blocking NMDA receptors. Memantine has been reported to help obsessive–compulsive disorder (OCD) as well as impulsive behaviors in open-label (44–48), single blind (49), and placebo-controlled studies (50, 51). In animal models, memantine has also been shown to decrease evidence of neuroinflammation (52).

Given this background, this article reviews studies that have been published to date, which have reported on the use of these Alzheimer’s medications in ASD individuals. Overall, this review demonstrates that there is significant scientific support for some of these medications in the treatment of core ASD symptoms, suggesting that further clinical trials may be helpful to help define the role for some of these medications for the treatment of individuals with ASD.

## Methods

### Search strategy

**Figure 1:**
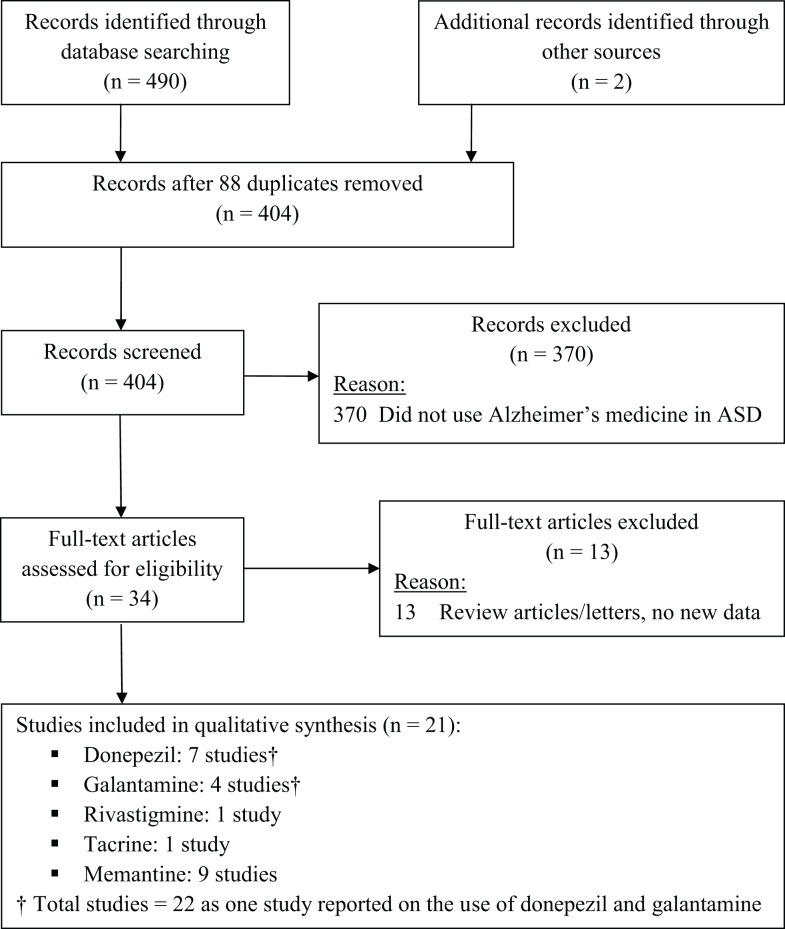
**Study flow chart**.

A computer-aided search of PUBMED [website (http://www.ncbi.nlm.nih.gov/entrez)] and Google Scholar databases from inception through the end of April, 2014 was conducted to identify pertinent articles using the search terms “autism,” “autistic,” “ASD,” “pervasive developmental disorder,” “PDD,” and “Asperger” in all combinations with the terms: “donepezil,” “galantamine,” “memantine,” “rivastigmine,” “tacrine,” “Alzheimer,” “cholinesterase inhibitor,” and “NMDA antagonist.” The references cited in identified articles were also searched to locate additional studies (two studies total). Figure 1 depicts the studies identified during the search process.

### Study selection

Studies were included if they: (1) involved individuals with ASD, and (2) administered a medication approved for Alzheimer’s disease to at least one individual with ASD. Articles that did not present new or unique data (such as review articles or letters to the editor) and animal studies were excluded. Studies on Rett syndrome and Childhood Disintegrative Disorder were also excluded. One reviewer screened titles and abstracts of all potentially relevant studies. After screening all records, 34 publications were identified and independently reviewed by both reviewers; 21 studies met inclusion criteria (see Figure 1). Within each section of this review, strengths and limitations of studies are discussed and recommendations for additional research are offered.

### Level of evidence ratings

Although we considered conducting a meta-analysis on identified treatments, the lack of standard outcomes and the limitations in study design prevented a meta-analysis of any identified treatment. As an alternative, we provide a grade of recommendation (GOR) for each treatment based on the level of evidence (LOE). Using a well-established scale (53), each study was individually assessed to determine the LOE, ranging from level 1 to 5 (Table 1). After assessing all identified studies for each treatment, a GOR ranging from A (solid evidence) to D (limited, inconsistent, or inconclusive evidence) was assigned (Table 2). Since a treatment could be a GOR of D for several reasons, we specified if the treatment received this rating because the evidence was a single case report or series (SC), was only based on bench research (BR), demonstrated an overall neutral effect (NE), or was found to be possibly detrimental (DE). If no studies were identified for a treatment, a GOR of N (no studies) was assigned. The overall ratings of Alzheimer disease medications used for ASD are found in Table 3. Table 4 lists the types of symptoms improved with each particular medication, while Table 5 compiles the side effects by medication.

**Table 1:** **Levels of evidence**.

Level	Description
1a	SR or meta-analysis of RCTs with homogeneity or Cochrane review with favorable findings
1b	Prospective high-quality RCT
2a	SR of cohort (prospective, non-randomized) studies with homogeneity
2b	Individual cohort (prospective, non-randomized) study or low-quality RCT
3a	SR of case–control (retrospective) studies with homogeneity
3b	Individual case–control (retrospective) study
4	Case-series or reports
5	Expert opinion without critical appraisal or based on physiology or bench research

*RCT, randomized controlled trial; SR, systematic review*.

**Table 2:** **Grade of recommendation**.

Grade	Description
A	At least one level 1a study or two level 1b studies
B	At least one level 1b, 2a, or 3a study, or two level 2b or 3b studies
C	At least one level 2b or 3b study, or two level 4 studies
D	Level 5 evidence, or troublingly inconsistent or inconclusive studies of any level, or studies reporting no improvements
N	No studies identified

**Table 3:** **Overall ratings of Alzheimer disease medications used for autism spectrum disorder**.

Medication	Uncontrolled studies positive (positive/total)	Controlled studies positive (positive/total)	Grade of recommendation
Donepezil	80% (4/5)	50% (1/2)	D – NE
Galantamine	100% (2/2)	100% (2/2)	B
Rivastigmine	100% (1/1)		D – SC
Tacrine	100% (1/1)		D – SC
Memantine	88% (7/8)	100% (1/1)	B

**Table 4:** **Improvements reported in studies of Alzheimer disease medications in ASD**.

Symptom	Donepezil	Galantamine	Rivastigmine	Tacrine	Memantine
Expressive language	X	X	X	X	X
Receptive language	X				X
Social interaction	X	X			X
Irritability	X	X		X	X
Hyperactivity	X	X		X	X
Attention	X	X			X
Eye contact	X	X		X	X
Emotional lability		X			
Repetitive or self-stimulatory behaviors					X
Motor planning					X
Disruptive behaviors					X
Obsessive–compulsive behaviors					X
Lethargy		X			
Overall ASD symptoms	X		X		
Increased REM sleep	X				

**Table 5:** **Reported side effects of Alzheimer disease medications in ASD**.

Side effect	Donepezil	Galantamine	Rivastigmine	Tacrine	Memantine
Irritability	X				X
Gastrointestinal problems	X				X
Verbal or behavioral regression	X	X			
Headaches		X			
Worsened behaviors					X
Irritability					X
Rash					X
Excessive sedation					X
Vomiting					X
Speech problems					X
Tremor	X				
Distractibility	X				
Increased seizures					X

## Results

### Donepezil

Seven studies reported on the use of donepezil in individuals with ASD, with five studies (71%) reporting improvements. Five studies were open-label or retrospective case-series. The first identified study was an uncontrolled, retrospective, open-label study of eight children (LOE 4) with autistic disorder (mean age 11.0 ± 4.1 years) and reported improvements in irritability and hyperactivity with donepezil (up to 10 mg/day) after at least 2 months of use; side effects included mild irritability (one patient) and gastrointestinal problems (nausea and vomiting in 1 patient) (54). Another uncontrolled, retrospective case-series (LOE 4) of eight children with PDD (ages 10–17 years) used donepezil (2.5–30 mg/day; mean 18 weeks of treatment) for ADHD-type symptoms and reported improvements in ADHD symptoms, communication, and socialization; one patient had to stop the medication due to side effects (tremor, irritability, and distractibility) (55). An uncontrolled, open-label study (LOE 4) of five children with ASD (ages 2.5–6.9 years) with deficits in REM sleep administered donepezil, which increased the percentage of REM sleep as measured by polysomnography after 1 month of treatment (56). In a case report (LOE 4) of a 5-year-old child with ASD, treatment with donepezil (5 mg at bedtime) over 6 weeks led to significant improvements in communication, eye contact, and hyperactivity (57). Finally, one case report (LOE 4) of three adults with autism treated with galantamine reported that the use of donepezil (dose not reported) led to verbal and behavioral regression in one adult (58).

Two studies were double-blind, placebo-controlled (DBPC) studies. The first DBPC study contained 43 children (LOE 1b) with autism (mean age 6.8 years) and administered donepezil or placebo over 6 weeks and reported improvements in expressive and receptive speech and a decrease in overall autistic behaviors in the treatment group; side effects included diarrhea and stomach cramps (59). In the second DBPC study of 34 children (LOE 1b) with ASD (age range 8–17 years), donepezil (dose 5–10 mg/day), or placebo was administered over 10 weeks, with a 10-week open-label trial of the medication for children in the placebo group who did not respond. No significant improvements were found in the donepezil group compared to the placebo group; no serious adverse events were observed (60).

Overall, the evidence for donepezil is inconsistent for improvements in ASD symptoms as one of the two DBPC studies was negative and one of the five case-series reported detrimental effects rather than beneficial effects of donepezil. As two case-series reported improvements in ADHD symptoms and one case-series demonstrated improvements in REM sleep, donepezil may have favorable effects on subsets of children with ASD who have these specific symptoms. Clearly, several studies have demonstrated favorable effects of donepezil, suggesting that further studies focused on specific symptoms may be warranted in the future.

### Galantamine

Four studies reported on the use of galantamine in individuals with ASD, with all four studies reporting improvements. Two of the studies were uncontrolled, open-label, or case-series (LOE 4). In the first case-series (LOE 4) of three adults with autism (21 to 42 years old), galantamine 4–16 mg/day was reported to increase expressive language and communication; however; one individual had a regression when put on donepezil (58). The second study was a 12-week, uncontrolled, open-label study (LOE 4) of galantamine in 13 children with autism (mean age 8.8 ± 3.5 years), which reported improvements in parent-rated social withdrawal and irritability on the Aberrant Behavior Checklist (ABC) and improvements in attention and emotional lability on the Conner’s Parent Rating Scale – Revised; eight patients were rated as improved on the Clinical Global Impression Scale (CGI); no significant adverse effect were found except for headaches in one child (61).

Two studies were DBPC. The first DBPC study of 20 children (LOE 2b) with autism (mean age 7.4 ± 3.2 years) reported a significant improvement with galantamine (dose not noted) compared to placebo on the ABC in irritability, eye contact, hyperactivity, and inappropriate speech; side effects were minimal (62). The second DBPC study of 40 children (LOE 1b) with autism (ages 4–12 years) reported that galantamine (up to 24 mg/day) for 10 weeks led to significant improvements in lethargy/social withdrawal and irritability on the ABC compared to placebo; side effects were similar in both groups (18).

Given that galantamine has been shown to improve both core and associated ASD symptoms in both open-label and DBPC studies (one high-quality and one of lower quality), it is given a GOR of B. Given this preliminary positive evidence, it is clear that large multicenter high-quality controlled trials should be conducted to provide efficacy data to further define the role of galantamine in the treatment of ASD.

### Rivastigmine

An open-label study of 32 children (LOE 4) with autism used rivastigmine (0.4–0.8 mg twice a day) for 12 weeks and reported improvements in expressive speech and overall autism symptoms (63). Given the limited number of studies on rivastigmine for the treatment of ASD, this treatment is given a GOR of D – SC. Because of the positive preliminary results and the fact that it was well tolerated, further larger open-label or blinded studies may be warranted for this treatment in ASD.

### Tacrine

An open-label study of three individuals (LOE 4) with ASD (mean age 17.4 ± 33.2 years) administered 20 mg of tacrine daily and reported mild improvements in irritability, hyperactivity, eye contact, and inappropriate speech as rated by combined parent and teacher scales (ABC); no significant side effects were reported (64). Given the limited number of studies on tacrine for the treatment of ASD, this treatment is given a GOR of D – SC. Because of the positive preliminary results and the fact that it was well tolerated, further studies may be warranted for this treatment in ASD to see if a wider number of children with ASD respond to this treatment.

### Memantine

Nine studies reported on the use of memantine in individuals with ASD, with eight studies (89%) reporting improvements. Eight studies were open-label or retrospective case-series. In the first study, Chez et al. (63) administered open-label (LOE 4) memantine (mean dose 8.1 mg/day, range 2.5–10 mg/day) in 30 children (mean age 8.92 years) with ASD and reported that for those treated more than 8 weeks (mean duration 18 weeks, range 8–40 weeks), 16 (53%) demonstrated significant improvements and 10 (33%) showed more mild improvements in attention, eye contact, language (expressive and receptive), repetitive behaviors, and motor planning; no significant side effects were observed (65). One case report (LOE 4) described the effects of memantine on a 23-year-old man with autistic disorder and observed improvements in disruptive behavior with memantine 10 mg/day over an 8-month period (66). An uncontrolled, open-label (LOE 4) study of 151 individuals with ASD (ages 2.58–26.33 years old) used memantine at a dose ranging from 2.5 to 30 mg and reported improvements in language, self-stimulatory behaviors, and social behavior; 22 patients (15%) had worsened behavior as a side effect (67).

An uncontrolled, open-label, and retrospective study (LOE 4) examined the effects of memantine (maximum dose 20 mg/day; duration of use 1.5–56 weeks) in 18 children with ASD (age 6–19 years) and reported improvements in social withdrawal and inattention; 7 patients (39%) had side effects including irritability (4 patients), rash (1 patient), excessive sedation and vomiting (1 patient), and an increase in seizures (1 patient); 4 patients had to discontinue memantine (68). An uncontrolled, open-label (LOE 4) study of four patients with ASD (mean age 17.4 ± 33.2 years) administered memantine 20 mg daily for 4 weeks and reported significant improvements on combined parent and teacher ratings on the ABC in irritability, hyperactivity, and inappropriate speech (*p* < 0.05 for all three); no side effects were reported (69). An uncontrolled, open-label study (LOE 4) used memantine (starting at 5 mg, increasing every 2 weeks up to 20 mg/day; mean final dose 18.3 ± 2.6 mg/day; mean use 34.7 ± 36.5 weeks, range 8–104 weeks) in six individuals with Fragile X and concomitant PDD (mean age 18.3 ± 3.8 years, range 13–22 years; four had autistic disorder and two had PDD). Four of the six patients were rated as “much improved” or “very much improved” on the CGI; non-significant improvements were observed on the ABC and SRS; two individuals experienced irritability, which led to drug discontinuation (70). A case report (LOE 4) of a 15-year-old boy with Asperger disorder, OCD, and Tourette disorder described the use of memantine (2.5 mg increasing to 10 mg/day) to treat the OCD symptoms and observed a significant reduction in OCD symptoms, including rituals and intrusive thoughts as well as improvements in social interaction; no significant adverse events were observed (71). One case study (LOE 4) reported stuttering and speech loss in two children with ASD who were taking memantine; in one child the speech improved with stopping the medication, while in the other child it improved while continuing the medication (72).

Only one study was DBPC and was a 10-week study of 40 children (LOE 1b) with ASD (ages 4–2 years). This study administered memantine (up to 15 mg/day if 10–40 kg; 20 mg/day if over 40 kg) compared to placebo and reported significant improvements on the ABC in irritability (*p* < 0.001), stereotypy (*p* < 0.01), and hyperactivity/non-compliance (*p* < 0.01); side effects were similar in both groups (73).

Given that the majority of clinical studies, including a DPBC study, provide positive evidence of improvements in both core and associated ASD symptoms, a GOR of B is provided for memantine for the treatment of ASD. Several studies have outlined adverse effects in certain patients with memantine treatment. Interestingly, memantine has been reported to both improve and worsen irritability. This suggests that there might be specific subgroups of children with ASD that respond optimally to memantine. Clearly larger, well-designed, and blinded studies are needed to further evaluate the efficacy of memantine in children with ASD as well as define the subgroups that might optimally respond to this medication.

## Discussion

This manuscript reviews the evidence for the use of medications which are FDA approved for Alzheimer’s disease in individuals with ASD. These medications target two neurotransmitter systems, acetylcholine and glutamate, which are both neurotransmitter systems with abnormalities associated with ASD. Overall five medications, four which target acetylcholine neurotransmission and one that targets glutamate neurotransmission, which are FDA indicated for Alzheimer’s disease have been used in individuals with ASD. To provide recommendations on the evidence for the potential usefulness of these medications for the treatment of ASD, we used an objective scale to rate the evidence for the utility of these medications for treating core and associated symptoms of ASD. Overall, we found that one medication that targets acetylcholine neurotransmission, galantamine, and one medication that targets glutamate neurotransmission, memantine, have reasonable evidence for the treatment of core and associated symptoms of ASD, although both require larger controlled studies to provide further efficacy data and define subgroups of individuals with ASD who may best respond to these treatments with limited adverse effects. Two medications, rivastigmine and tacrine, both of which target the acetylcholine neurotransmitter system, have only preliminary uncontrolled studies to support their use, so further studies need to be performed before recommendations can be made. One medication that targets acetylcholine, donepezil, has several studies investigating its use in individuals with ASD but the results of some of the studies, particularly the DBPC studies, are inconsistent, making recommendations difficult at this time, although in certain subgroups (ADHD symptoms or REM sleep problems) it might be of use.

Given the fact that there is no FDA approved medication for the core symptoms of ASD and considering the limited proven effective treatments for ASD, studies are needed to identify novel treatments. Because several of the medications reviewed here show promising evidence for effectiveness for treating core and associated ASD symptoms, such medications should undergo further study in clinical trials to confirm their effectiveness for treating individuals with ASD.

## Conflict of Interest Statement

The authors declare that the research was conducted in the absence of any commercial or financial relationships that could be construed as a potential conflict of interest.
